# A Tale of Two Signals: AR and WNT in Development and Tumorigenesis of Prostate and Mammary Gland

**DOI:** 10.3390/cancers9020014

**Published:** 2017-01-27

**Authors:** Hubert Pakula, Dongxi Xiang, Zhe Li

**Affiliations:** 1 Division of Genetics, Brigham and Women’s Hospital, 77 Avenue Louis Pasteur, Room 466, Boston, MA 02115, USA; hpakula@partners.org (H.P.); dxiang@bwh.harvard.edu (D.X.); 2 Department of Medicine, Harvard Medical School, Boston, MA 02115, USA

**Keywords:** androgen receptor, AR, WNT, prostate, prostate cancer, castration-resistant prostate cancer, CRPC, mammary gland, breast cancer

## Abstract

Prostate cancer (PCa) is one of the most common cancers and among the leading causes of cancer deaths for men in industrialized countries. It has long been recognized that the prostate is an androgen-dependent organ and PCa is an androgen-dependent disease. Androgen action is mediated by the androgen receptor (AR). Androgen deprivation therapy (ADT) is the standard treatment for metastatic PCa. However, almost all advanced PCa cases progress to castration-resistant prostate cancer (CRPC) after a period of ADT. A variety of mechanisms of progression from androgen-dependent PCa to CRPC under ADT have been postulated, but it remains largely unclear as to when and how castration resistance arises within prostate tumors. In addition, AR signaling may be modulated by extracellular factors among which are the cysteine-rich glycoproteins WNTs. The WNTs are capable of signaling through several pathways, the best-characterized being the canonical WNT/β-catenin/TCF-mediated canonical pathway. Recent studies from sequencing PCa genomes revealed that CRPC cells frequently harbor mutations in major components of the WNT/β-catenin pathway. Moreover, the finding of an interaction between β-catenin and AR suggests a possible mechanism of cross talk between WNT and androgen/AR signaling pathways. In this review, we discuss the current knowledge of both AR and WNT pathways in prostate development and tumorigenesis, and their interaction during development of CRPC. We also review the possible therapeutic application of drugs that target both AR and WNT/β-catenin pathways. Finally, we extend our review of AR and WNT signaling to the mammary gland system and breast cancer. We highlight that the role of AR signaling and its interaction with WNT signaling in these two hormone-related cancer types are highly context-dependent.

## 1. Introduction

For the men in the United States, prostate cancer (PCa) is not only one of the most commonly diagnosed cancers, but also one of the most predominant causes of death from cancer [1,2]. The American Cancer Society estimates that in 2016, there will be 180,890 newly diagnosed cases and 26,120 deaths due to PCa in the United States, making it the second leading cause of cancer death in men [3]. Since the prostate gland development depends on androgens and androgen receptor (AR) signaling [4,5], human PCa initially responds to androgen-deprivation therapy (ADT) [6,7]. However, the cancer often reappears, and is accompanied by rising levels of serum prostate-specific antigen (PSA) [8,9]. PSA (KLK3) is encoded by an androgen-dependent gene, and increased expression of PSA in an environment of castrate levels of circulating androgens indicates that adaptive androgen signaling has emerged in the tumor [10,11]. Accordingly, in the majority of cases, an initially hormone-sensitive PCa will evolve to a lethal castration-resistant prostate cancer (CRPC) [12,13,14,15]. The underlying molecular basis for how PCa cells escape from the growth control by exogenous androgens remains poorly understood. Recent studies, however, pointed to the AR and its actions as a key factor in many CRPCs, despite the reduction in circulating testosterone. The mechanisms involved in this change include increased expression and stability of the AR protein, activating mutations in this receptor that alter its ligand specificity, and changes in the expression of transcriptional co-regulators of the AR [16,17]. In addition, AR and its cognate ligands interact with potent oncogenic systems, such as WNT signaling, to elicit changes in cellular adhesion and oncogenesis [18,19,20,21].

WNT signaling is an evolutionary highly conserved signaling system throughout the eukaryotic kingdom. During embryonic and postnatal development, WNT signaling controls many cellular processes, including proliferation, survival and differentiation [22,23,24,25,26]. Deregulation in WNT signaling leads to an imbalance of such processes, often resulting in aberrant development or disease [27,28]; in particular, deregulated WNT signaling is common in human cancers, including malignancies of the intestine [29,30,31], liver [32,33,34,35], skin [36,37], breast [38,39,40,41] and prostate [42,43].

The term Wnt is an amalgam of Wg and Int [44], as the genes *Wingless* (*Wg*) and *integration 1 (Int1)* are homologues in *Drosophila* and mouse, respectively [45,46]. *Wg* was genetically characterized as a segment polarity gene in *Drosophila* in 1980 by Nüsslein-Volhard and Wieschaus [47]. The proto-oncogene *Int1* was first identified in 1982 by Nusse and Varmus as a preferential site for proviral integration of the mouse mammary tumor virus (MMTV) in a mouse mammary cancer model [48]. Since the identification of *Wnt1*, genome sequencing has revealed the existence of 19 *Wnt* genes in mammals. All WNT proteins share common features that are essential for their function, including a signal peptide for secretion, many potential glycosylation sites, and WNT ligands interact with seven-pass transmembrane receptors of the Frizzled (FZD) family and/or single-pass transmembrane co-receptors, such as lipoprotein receptor-related protein 5/6 (LRP5/6), ROR2, and RYK [49,50,51,52,53,54]. Co-factors such as R-spondin and Wise also take part in WNT-receptor complex activity [55,56,57]. R-spondin/LGR (leucine-rich repeat-containing G-protein coupled-like receptor) complexes and WNT ligands directly interact with FZD-LRP-receptor complexes on target cells to activate downstream signaling. This leads to the activation of various intracellular signaling cascades that can be cross-connected or act independently. The intracellular signaling activated by WNT proteins is organized into two categories: canonical and non-canonical. Canonical WNT signaling is often referred to as the WNT/β-catenin pathway, as it relies on β-catenin-dependent transcriptional activation triggered by WNT-stimulated signals. In contrast, non-canonical WNT pathways, including the WNT/Ca2^+^ (calcium) and WNT/JNK (c-Jun N-terminal kinase), WNT/Rho pathways, are β-catenin-independent and activate a variety of downstream intracellular signaling cascades [26,58,59,60]. These mechanisms have been the subject of numerous reviews [22,23,24,25,26], and therefore will only be briefly described here.

In this review, we will discuss the multifaceted manner with which both the canonical and non-canonical WNT pathways influence and modulate AR signaling in CRPC development. We will consider the possible therapeutic application of drugs that target both pathways. We will also discuss these under the context of recurrent mutations in both pathways identified from PCa genomes. Finally, we will extend our review of these two pathways to the mammary gland system and breast cancer.

## 2. An Overview of the Canonical and Non-Canonical WNT Signaling Pathways

**Figure 1:**
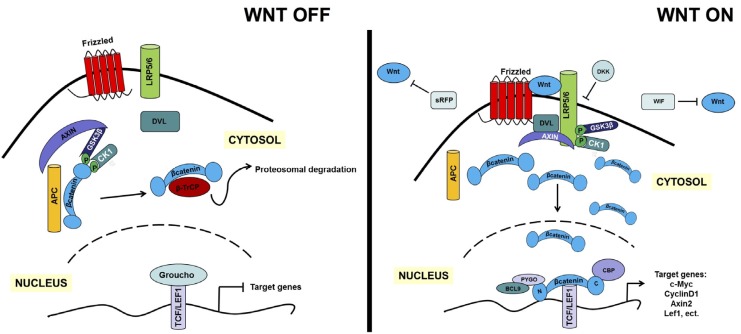
Schematic diagram of the canonical WNT signaling pathway. (left panel) the “WNT-Off” state: In the absence of a WNT signal, β-catenin levels in the cytoplasm are kept low through proteosomal degradation induced by the β-catenin destruction complex. Grouchos (transcriptional co-repressors) interact with TCF/LEF proteins and prevent the expression of WNT target genes. (right panel) the “WNT-On” state: When WNT ligands bind to their receptors Frizzled (FZD) and LRP5/6, the receptor complex can recruit components of the β-catenin destruction complex, resulting in accumulation of β-catenin in the cytoplasm. β-catenin will then translocate into the nucleus, replace Grouchos and recruit transcriptional co-activators to form the transcription complex with TCF/LEF proteins, which eventually promote expression of the WNT target genes.

The known molecular components and the cascade of the canonical WNT signaling pathway are summarized in Figure 1. Canonical WNT signaling strictly controls the level of the cytoplasmic protein β-catenin. β-Catenin, encoded by the *CTNNB1* gene [61], is a member of the armadillo family of proteins. β-Catenin consists of an N-terminal region of 149 amino acids, followed by a central domain of 515 residues composed of 12 armadillo repeats, and a C-terminal region of 108 residues [62]. The N-terminal region contains phosphorylation sites recognized by GSK3β and CK1α and an α-catenin binding site, whereas the C-terminal region works as a transcriptional co-activator-binding domain (CBD) that interacts with histone modifiers such as histone acetyltransferases CBP/P300 [63]. β-Catenin has dual functions. It acts as a transcription cofactor with the T cell factor/lymphoid enhancer factor (TCF/LEF) in the WNT pathway [64,65,66,67]. It is also a structural adaptor protein that binds E-cadherin and α-catenin through its Armadillo repeats and N-terminal domain, respectively (E-cadherin is a core transmembrane adhesion protein, and α-catenin is a protein that binds actin and other actin-regulators) [68,69,70,71,72]. The multifaceted functions of β-catenin are regulated by three cellular pools of this molecule that are under strict regulation: a membrane pool of cadherin-associated β-catenin, a cytoplasmic pool, and a nuclear pool [73]. Canonical WNT signaling works in the following fashion: in the absence of WNT signals, β-catenin is efficiently captured by scaffold proteins, the AXINs, which are present within a destruction complex containing glycogen synthase kinase (GSK3β), adenomatous polyposis coli (APC) and the casein kinase-1 (CK1). The resident CK1 and GSK3β protein kinases sequentially phosphorylate conserved serine and threonine residues in the N-terminus of the captured β-catenin, generating a binding site for an E3 ubiquitin ligase. Ubiquitination targets β-catenin into proteasomes for rapid degradation [74,75,76,77]. Therefore, in the absence of WNT, cytoplasmic β-catenin levels remain low, and the transcription factors LEF1 and TCF interact with Grouchos in the nucleus to repress WNT pathway-specific target genes [78,79]. In contrast, upon the interaction of canonical WNT ligands to its receptors, FZD, and co-receptor, LRP5/6, the destruction complex is disassembled through phosphorylation of LRP5/6 by CK1γ and binding of AXIN to LRP, which prevents β-catenin degradation [80,81]. The inactivation of the destruction complex allows cytoplasmic stabilization and translocation of β-catenin to the nucleus, where it interacts with members of the TCF/LEF family [64,65,66] and converts the TCF/LEF proteins into potent transcriptional activators. It achieves this by displacing Grouchos [82] and by recruiting other co-activators such as B-cell lymphoma 9 (BCL9) [83,84], Pygopus [85,86], CREB-binding protein (CBP) [87,88] or Hyrax [89], ensuring efficient activation of WNT target genes encoding c-Myc [90], Cyclin D1 [91,92], urokinase-type plasminogen activator (uPA) [93], CD44 [94], Cox-2 and Cox-9 [95], and the *AR* gene [96,97], as well as genes that encode key components of the WNT pathway (e.g., FZDs, DKKs (Dickkopf), LRPs, AXIN2, β-TrCP and TCF/LEF) (Figure 1). These WNT target genes then influence cell cycle regulation, stem cell function and development, as well as invasion and metastasis of cancer cells. For an updated overview of the WNT pathway and its target genes, see the WNT homepage at http://www.stanford.edu/group/nusselab/cgi-bin/wnt/.

In addition to promoting the WNT activity, a series of biochemical experiments indicated that R-spondins (RSPOs) are able to synergize with the WNT pathway in the presence of canonical WNT ligands [98]. Similar to the WNT proteins, RSPOs are also cysteine-rich. However, unlike WNTs, the cysteine residues found in RSPOs are organized into two adjacent furin-like domains, which have been suggested to be sufficient for inducing β-catenin stabilization [98]. Recently, LGR4, LGR5 and LGR6, three closely related LGR proteins, have been identified as receptors for RSPOs. *LGR5* is a WNT target gene and although originally discovered as an intestinal stem cell marker [99], it has also become an ideal candidate marker for understanding stem cell and cancer biology of other epithelial cell types in mice and human [56,99,100,101]. The LGR5 protein had previously been identified as an orphan receptor, among LGRs. The LGR family is defined by a large extracellular N-terminal domain composed of a string of leucine-rich repeat units, a 7-transmembrane domains (7TM) and a cytoplasmic region. Specifically, LGR5, together with LGR4 and LGR6, belong to the B-class LGRs [100,102]. Close relatives are the LGRs for the follicle stimulating hormone (FSH), the luteinizing hormone (LH) and the thyroid-stimulating hormone (TSH), which are true G-protein coupled receptors. Recently, it was found that instead of binding hormones, the LGR4/5/6 receptors interact with RSPOs and do not activate G-proteins; instead, they promote WNT/β-catenin signaling. Specifically, the interaction of RSPOs and LGR5 has been assessed in cell surface binding assays, cell-free co-immunoprecipitation and tandem affinity purification mass spectrometry [55,102,103]. As their potentiating ability depends on the presence of a WNT ligand, the WNT secretion machinery can thus indirectly affect their role on WNT signaling.

The activation of canonical WNT signaling can also be blocked by extracellular proteins. These include the sFRP family (secreted frizzled related protein; sFRP1, 2, 4, and 5) [104], WIF (Wnt inhibitory factor) [105], the DKK family of proteins (DKK1–4 and DKKL1) [106], and the cysteine knot family proteins SOST [107] and WISE [108]. These soluble inhibitors bind to WNT, the FZD receptor in the case of sFRP, or to the co-receptor LRP5/6 in the case of DKK1 and SOST/WISE, thereby interfering with ligand–receptor complex formation and blocking WNT signaling [109].

While the canonical WNT signaling pathway has been extensively dissected biochemically and at the molecular level, non-canonical WNT signaling has been less focused on. The best characterized non-canonical WNT pathways include the WNT/Ca2^+^ pathway, which was first described in vertebrates [58], and the planar polarity pathway (PCP), which was first identified in *Drosophila* [110]. Other non-canonical pathways include WNT/JNK and WNT/Rho signaling [111].

In the WNT/Ca2^+^ pathway, the interaction of non-canonical WNT ligands and receptors recruits Dishevelled (DVL) and G protein, which activates phospholipase C (PLC), leading to production of 1,2-diacylglycerol (DAG); 1,2-DAG then activates protein kinase C (PKC), and inositol 1,4,5-triphosphate (IP3), thereby triggering intracellular calcium release from the endoplasmic reticulum [112,113]. Calcium release activates calcineurin (CNA) and Ca^2+^/calmodulin-dependent protein kinase II (CAMKII), which increase expression of nuclear factor of activated T cells (NFAT)-dependent genes and inhibit canonical WNT signaling through nemo-like kinase (NLK), respectively [114,115]. Activated NFAT may boost the expression of several genes in neurons, cardiac and skeletal muscle cells, prostate, and pro-inflammatory genes in lymphocytes [116,117,118]. In the WNT-PCP pathway, FZD receptors activate a signaling cascade that involves the small GTPases Rho and Rac and c-Jun N-terminal kinase (JNK) [119]. In contrast to calcium-regulated non-canonical signaling, WNT/JNK signaling uses ROR2-dependent circuitry to activate downstream effectors of the activating protein-1 (AP-1) family of transcription factors [59,60]. In addition, a new β-catenin-independent aspect of WNT signaling was recently reported in proliferating cells: WNT signaling was found to peak at the G2/M phase of the cell cycle to produce the so-called WNT-dependent stabilization of proteins (WNT/STOP) [120,121]. This appears to be a dominant mode of WNT signaling in several cancer cell lines, where it is required for cell growth. Of note, boundaries of both canonical and non-canonical WNT pathways are not stringent and there are considerable degrees of overlapping between them [122].

## 3. WNT Signaling in Prostate Development and Stem Cells

In both human and rodents, the prostate gland surrounds the urethra at the base of the bladder and functions by contributing secretory proteins to the seminal fluid. In men, the prostate gland is a walnut-sized tissue with a zonal architecture, corresponding to central, periurethral transition, and peripheral zones, together with an anterior fibromuscular stroma [123]. Importantly, the outermost peripheral zone occupies the most volume, and harbors the majority of prostate carcinomas. In contrast, benign prostatic hyperplasia (BPH), a common nonmalignant condition found in older men, arises from the transition zone [124]. Unlike the human prostate that is a compact gland, the mouse prostate includes four paired lobes situated circumferentially around the urethra: anterior (AP), dorsal (DP), lateral (LP), and ventral (VP) prostate. The DP and LP are sometimes collectively referred to as the dorsolateral lobes of the prostate (DLP). At birth, each lobe of the VP consists of 1–3 main ducts with secondary and tertiary branches, whereas the more complex DLP initially has 9–12 unbranched proximal main ducts on each side [125,126].

In all species, formation of the prostate gland initiates during embryogenesis. During mid-gestation, the primitive urogenital sinus (UGS) is separated from the terminal region of the hindgut through division of the cloaca by the urorectal septum. The most rostral region (vesiculo-urethral part) of the primitive UGS forms the urinary bladder, whereas the most caudal region (phallic part) forms the penile urethra. The prostate gland originates from a sub-compartment of the lower urogenital tract (LUT), known as the definitive UGS [127,128]. The endodermal UGS is surrounded by embryonic connective tissue called urogenital sinus mesenchyme (UGM). Prostate development, growth and function is androgen dependent; however, other steroid receptors, such as estrogen receptors (ER) and retinoid receptors (RARs and RXRs), also contribute to prostate morphogenesis and differentiation. Prior to sexual differentiation of the UGS, UGM expresses AR in both sexes and thus acquires the capacity to undergo masculine development [129,130,131]. Over 30-year of research by Cunha and colleagues has shown that an AR-dependent signal from the urogenital mesenchyme is required for prostate formation, while AR is not initially required in the urogenital epithelium (UGE) for prostate organogenesis, but is subsequently necessary for epithelial differentiation and secretory protein expression [124,132,133,134]. In mouse, the prostatic ducts start to form after embryonic day 17 (E17) as solid epithelial buds formed from the UGE that invades the surrounding UGM [126]. During perinatal and neonatal development, prostatic buds undergo primary, secondary, and tertiary branching morphogenesis in a pattern unique to each pair of the DP, VP, LP, and AP lobes in rodents [125]. The rate of new VP ductal tip formation in Balb/c mice, a hallmark of branching morphogenesis, peaks at about postnatal day 5 (P5). Concurrent with branching morphogenesis, epithelial buds canalize in a proximal to distal direction along the developing ducts, giving rise to two distinct cell layers: a superficial layer of secretory columnar luminal epithelium lining prostatic ducts and a deep layer of basal epithelium including the rare neuroendocrine cells [135,136]. Basic prostatic architecture is established during puberty, upon an androgen-driven increase in prostate gland size. After that the prostatic epithelium reorganizes into a layer of outer cuboidal basal cells and inner tall columnar luminal cells. Human prostate development proceeds by a similar series of morphogenetic events, but gives rise to a mature prostate that contains a single capsulated structure divided into peripheral, central, and transitional zones. The basal cells express cytokeratins 5 and 14, and p63 and are localized along the basement membrane, but express AR at low or undetectable levels [137]. The luminal cells express cytokeratins 8 and 18 as well as high levels of AR [138]. In humans, mature luminal cells constitute the exocrine part of the prostate and secrete PSA and PAP (prostate acid phosphatase) [139,140]. The third epithelial cell type in the prostate is the androgen-independent neuroendocrine cell, which makes up only a small proportion of the prostate epithelial cells and is characterized by expression of functional markers such as chromogranin A and synaptophysin [141,142]. In addition, intermediate or transit-amplifying cells that express both the basal and luminal lineage markers are detectable during the developmental stage, under pathological conditions in adults, or when prostate epithelial cells are cultured in vitro [137,143,144,145,146].

The use of transgenic mice combined with molecular analyses have demonstrated the importance of several developmental signaling pathways during prostate organogenesis, including bone morphogenetic protein (BMP), transforming growth factor beta (TGFβ), Notch, sonic hedgehog (SHH), and WNT pathways [147]. Evidence that WNT signaling is involved in prostate morphogenesis comes from studies by Zhang et al. [148]. By creating six LongSAGE libraries at three key stages of prostate organogenesis: E16.5 UGS (i.e., a stage just before the first prostate buds are formed), P0 prostates (i.e., a stage when branching morphogenesis has begun), and 12-week adult prostates (i.e., a time of relative growth quiescence), Zhang and colleagues evaluated sex and cell-type specific genes associated with prostate induction and found expression changes of multiple WNT-related genes, such as *Sfrp2*, *Wnt4*, *Wnt5a*, *Wnt11*, *Fzd1*, *Fzd7*, *Fzd10*, *Lrp5*, *Axin1*, *Lef1*, *Nkd1*, and *RhoA* [148,149]. Accordingly, in vivo studies using *Sfrp1-*overexpressing transgenic mice and *Sfrp1-*null mice confirmed that this WNT modulator stimulates prostate branching morphogenesis, epithelial cell proliferation and secretory gene expression [150]. Additionally, in vitro studies by Prins and colleagues showed that the WNT signaling inhibitor DKK1 also stimulated growth and branching of cultured newborn rat VP lobes over a four-day period, suggesting that canonical WNT signaling suppresses prostate growth [147]. This was supported by another recent study where WNT3A, a canonical WNT ligand, reduced ductal branching of cultured neonatal rodent (rat) prostates and active canonical WNT signaling in epithelial progenitor cells maintaining their undifferentiated state [151]. In addition, *Wnt5a* was found to be indispensable during the UGS development. High levels of *Wnt5a* expression has been observed at the distal tips and along the centro-distal periductal mesenchyme during the period of postnatal branching morphogenesis, with a rapid decline thereafter in the VP but not the DP and LP [152]. Another study further demonstrated that loss of *Wnt5a* impeded buds branching during morphogenesis [153].

β-Catenin has been identified in both epithelial and mesenchymal structures that undergo a budding program; its activation is necessary and/or sufficient for specification of hair follicle, mammary gland and tooth buds [154,155,156]. Of note, an absolute requirement for this protein has been shown in prostatic induction. While conditional expression of a constitutively active form of β-catenin in developing prostate epithelium prevents epithelial differentiation [136,157], conditional deletion of the β-catenin gene (*Ctnnb1*) in the mouse prostate during embryonic stages results in significantly decreased prostatic budding and abrogates prostatic development [158]. Furthermore, a recent study by Mehta et al. demonstrated the importance of WNT-activators RSPOs in murine prostatic bud formation [136]. By in situ hybridization (ISH), Mehta et al. unveiled the expression pattern of *R-spondin1-4* (*Rspo1-4*) in developing and neonatal mouse LUT. They found that *Rspo3*, together with *Wnt4*, *Wnt10b*, *Wnt11* and *Wnt16*, appear to be more abundant in male versus female UGS and they stimulate prostatic development [136].

Although development of the adult prostate is largely completed at puberty, it must possess a mechanism to assure the homeostasis of its epithelium. To achieve this, prostate, similar to other epithelial organs, sets aside a life-long reservoir of somatic stem cells that retain self-renewal. The regenerative capacity of prostate epithelial stem cells (PSCs) has been shown in the experiment with repeated rounds of androgen ablation and restoration; thus PSCs are androgen-sensitive but not dependent, are capable of self-regeneration, and give rise to transit-amplifying cells that differentiate into various specialized epithelial cells of the prostate [159]. To date, the best approach to identify and characterize murine and human PSCs is to combine flow cytometry with functional assays, such as genetic lineage tracing experiments, tissue culture and renal capsule implantation. Specifically, first prostate epithelial cells are fractionated based on their surface antigenic profiles and then functional assays are used to determine whether different subpopulations possess stem cell activity or not. Based on this approach, the basal cell subpopulation appeared to be bipotent, i.e., capable of generating both luminal and basal lineages, thus indicating that basal cells have stem cell-like potential [160,161,162]. Independent studies by the two laboratories of Witte and Wilson showed that makers such as CD49f, Trop2 and CD166 could enrich prostate cells for the PSC activity among the Sca-1^+^ cells [145,163,164,165,166,167]. Similarly, Richardson et al. isolated human prostate cells expressing a stem cell marker CD133 and showed that α2β1integrin^+^CD133^+^ basal cells also correspond to an enriched stem cell fraction in the human prostate epithelium [168]. Finally, Leong et al. reported successful regeneration of prostatic tissues from single Lin^−^Sca-1^+^CD133^+^CD44^+^CD117^+^ cells, which are predominantly basal in mice and are exclusively basal in humans [169]. In addition to the cellular hierarchy of the prostatic epithelium in mice, Wang et al. showed in the lineage tracing experiments that rare luminal cells (i.e., castration-resistant *Nkx3-1* expressing cells (CARNs)) are bipotential and can self-renew in vivo [170]. Nevertheless, a full understanding the properties of prostate luminal epithelial cells has been hampered by the lack of suitable in vitro model systems. In comparison to the basal epithelial cells, luminal epithelial cells are indeed more sensitive for tissue dissociation, after which they fail to survive in explant culture or grafts [170,171]. To circumvent this technical difficulty, three-dimensional (3D) organoid culture was developed recently [172]. By using testosterone-responsive culture conditions, Karthaus et al. confirmed that human prostate luminal cells have potential to generate both basal and luminal lineages. Moreover, they showed that basal and luminal cells can each generate a complete multilayer prostate organoids, suggesting that both lineages have stem cell-like potentials [173]. Of note, the 3D organoid system, although mimicking a testosterone-naïve environment for the single stem cells, relies also on the addition of LGR4/5 ligand R-spondin1, a potent WNT/β-catenin agonist. This might shed a new light on the role of WNT activity in the maintenance and expansion of PSCs and their progeny. In fact, evidence of the importance of WNT activity in the maintenance of PSCs and their progeny was provided in two consecutive studies by the laboratory of Wilson; in one study, Blum et al. determined the transcriptional profiles of four populations of prostate cells: (i) urogenital epithelium from 16-day embryos, that represent fetal PSCs; (ii) Sca-1^High^ cells, enriched in adult PSCs; (iii) Sca-1^Low^ cells, that represent transit-amplifying cells; and (iv) Sca-1^Negative^ cells representing terminally differentiated population with no regenerative potential [174]. Upregulation of WNT signaling was observed in both fetal and adult PSCs. However, WNT signaling acts differently in these two populations, as the fetal PSC population is highly proliferating, whereas the adult PSC population is quiescent [174]. In another work, the same group reported that WNT receptors such as FZD6 and ligands such as WNT2 and WNT4 also control the stem cell niche activity [175]. Similarly, other WNT ligand has been shown to be critical in controlling self-renewal of PSCs in a prostasphere culture system [94]. Interestingly, activation of canonical WNT pathway through WNT3A results in a significant increase of the expression of nuclear β-catenin [94]. This is consistent with other reports showing that WNT3A signaling can preserve an undifferentiated phenotype in CD133^+^ human cord blood-derived cells [176] and it supports embryonic stem cell self-renewal [177]. Furthermore, the importance of β-catenin in the self-renewal of Lin^−^Sca^−^CD49f^high^ mouse prostate stem and progenitor cells has been provided in the study by Lukacs et al. [178]. This group reported that cells expressing the BMI-1 (polycomb group) protein require constitutively active β-catenin for increased self-renewal. This suggests that BMI-1 may be a mediator of WNT/FZD signaling in normal PSCs [178].

## 4. An Overview of AR and AR Signaling

The most critical molecular component of the androgen signaling pathway is the AR protein. Upon activation by androgens, AR mediates transcription of target genes that modulate growth and differentiation of prostate epithelial cells. AR plays a vital role in the development of male reproductive organs. Of note, its dysregulation contributes to the male pattern of baldness, development of prostatic hyperplasia, and later in life to PCa.

**Figure 2:**
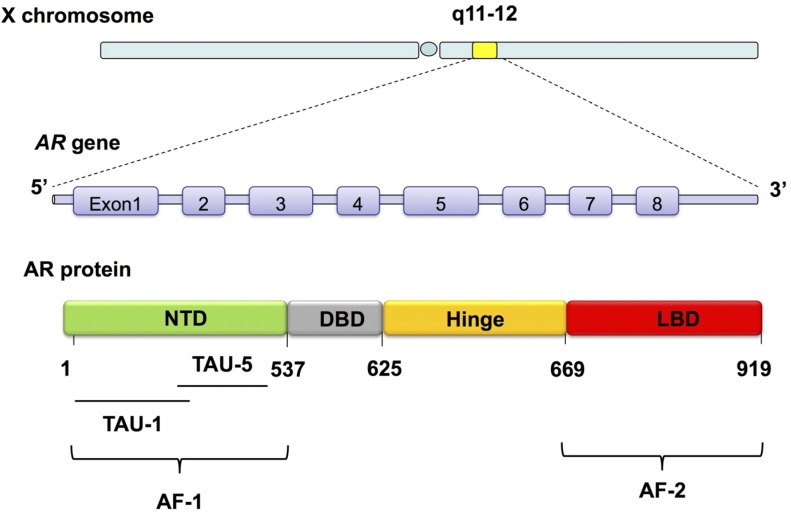
Schematic representation of the androgen receptor (AR) gene and protein, with indications of its specific motifs and domains. The *AR* gene is located on human X chromosome and is composed of 8 exons. The domains and motifs in the AR protein include: the N-terminal domain (NTD), the DNA-binding domain (DBD), the hinge region, and the ligand-binding domain (LBD), as well as the activation function 1 (AF-1) domain and the activation function 2 (AF-2) domain, and two transcription activation units (TAUs): TAU-1 and TAU-5.

The *AR* gene is located on chromosome Xq11-12. It consists eight exons that encode an 110 kDa nuclear receptor that is a unique member of the nuclear steroid receptor gene family (Figure 2) [179,180]. The AR protein has four functional domains (Figure 2). The N-terminal domain (NTD) is the most variable and least conserved domain; it is needed to form a transcriptionally active molecule. Precisely, the NTD contains the activation function 1 (AF-1) domain that includes two overlapping transcription activation units (TAUs): TAU-1 (amino acids 1–370), which supports AR transcriptional activity upon stimulation by full agonist, and TAU-5 (amino acids 360–528), which confers a constitutive activity to the AR in the absence of its ligand-binding domain (LBD) (Figure 2) [181,182,183]. Next to the NTD lies the DNA-binding domain (DBD), which is the most conserved region in this protein. This DBD consists of two zinc finger modules that are responsible for binding to the hormone response elements [184,185]. The carboxy-terminal end of AR contains the LBD and the activation function 2 (AF-2) domain [183]. Lastly, the region between the DBD and LBD of AR is termed the hinge region (HR) (Figure 2). It provides the main portion of the nuclear translocation signal and regulates the transactivation potential as a result of posttranslational modifications. Interestingly, it serves as an integrator for signals coming from different pathways [185].

**Figure 3:**
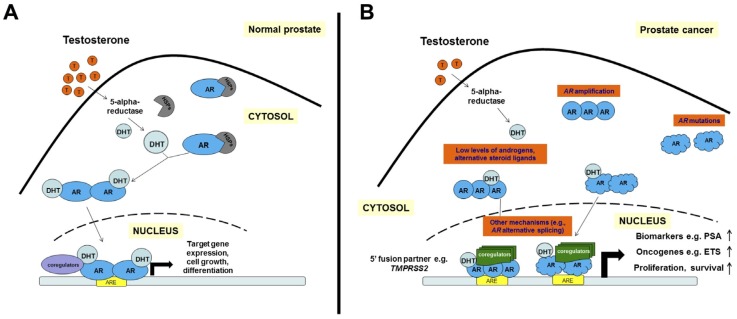
Schematic representation of AR signaling in normal prostate tissue and prostate cancer. (**A**) The AR is complexed to heat shock proteins (HSPs), principally HSP90, in the absence of steroid hormones. Upon binding to dihydrotestosterone (DHT), AR dimerizes and translocates to the nucleus. In the nucleus, AR binds to DNA via the androgen-responsive element (ARE). This occurs both by direct binding to DNA and by association with other transcription factors and co-regulators, leading to expression of its target genes that control growth and differentiation of prostate cells; (**B**) In PCa cells, AR signaling is maintained through other mechanisms such as *AR* amplification, *AR* mutations, or *AR* alternative splicing. AR can also be transactivated in the absence or under very low levels of androgens. In the nucleus, AR can drive expression of oncogenes such as those encoding the ETS transcription factors (e.g., ERG, ETV1), as a consequence of gene rearrangements (e.g., *TMPRSS2-ERG* gene fusion); it also controls expression of its target genes that support proliferation and survival of PCa cells.

In mammalian cells, AR is sequestered in the cytoplasm and is bound to heat shock protein complex consisting of Hsp70 (hsc70), Hsp40 (Ydj1), Hop (p60), Hsp90 and p23. The main role of this complex is to maintain AR in a conformation capable of ligand binding and to protect it from proteolysis [182,186,187,188]. Upon binding to testosterone or dihydrotestosterone (DHT), the chaperone heterocomplex mediates AR translocation to the nucleus (Figure 3). In the canonical genomic pathway, once in the nucleus, AR, as a homodimer, interacts with androgen response elements (ARE); by recruiting co-regulators to form a pre-initiation complex and together with the basal transcriptional machinery, it initiates transcription of its target genes (Figure 3A) [189,190,191]. Of note, nuclear targeting of AR is influenced by its HR, where a deletion markedly reduces ligand-induced nuclear translocation, but does not totally block signaling [192,193,194]. Subsequently, loss of bound ligand allows the nuclear export signal (NES) to coordinate AR shuttling to the cytoplasm where AR can be tethered again to cytoskeletal proteins in preparation for ligand binding [195,196].

Regulation of the AR activity occurs, in part, by posttranslational modifications, such as phosphorylation at several serine residues with or without a bound ligand [197]. Precisely, AR is phosphorylated at serine residues (Ser80, Ser93 and Ser641) that are believed to function by protecting AR from proteolytic degradation [196,198]. Degradation of AR plays a pivotal role in the regulation of AR function. AR is a direct target for MDM2-mediated ubiquitylation and proteolysis [199]. The NEDD4 ubiquitin ligase recruiting protein PMEPA1 may also play important roles in this pathway [200,201].

## 5. The Emergence of Castration Resistance

Although the preferred ligand for AR is DHT (Figure 3A), it has been reported that mutations frequently detected in both human PCa and in PCa cell lines may alter the ligand specificity of AR, leading to its promiscuous activity in the presence of alternative steroid ligands that do not bind to the wild-type AR [202,203]. In addition to mutations of *AR* found in PCa, important recent studies have shown that AR can drive expression of oncogenes such as those encoding the ETS transcription factors (e.g., ERG, ETV1) as a consequence of gene rearrangements [204]. The most common form of these rearrangements creates a *TMPRSS2-ERG* gene fusion, resulting in expression of an N-terminally truncated ERG protein under the control of the androgen-responsive promoter of *TMPRSS2* (Figure 3B) [204,205]. Furthermore, a recent whole-genome chromatin immunoprecipitation (ChIP) analysis showed that ERG could bind to AR downstream target genes and disturb AR signaling in PCa cells through epigenetic silencing [206]. By characterizing human PCa cell lines and knockin mouse models ectopically expressing ERG or ETV1, we demonstrated that ERG negatively regulates the AR transcriptional program, whereas ETV1 cooperates with AR signaling by favoring activation of the AR transcriptional program [207].

Prostate gland development and PCa are critically dependent on AR signaling. The ADT remains the most widely used treatment for patients with advanced PCa. In fact, androgen deprivation causes reduced AR expression, apoptosis and decreased tumor cell volume; however most PCas eventually develop the capacity for recurrent growth in the absence of testicular androgen (i.e., CRPC) [208,209,210]. The postulated mechanisms to explain the emergence of CRPC can be separated into three general categories, most of which center on AR signaling, including *AR* amplification, *AR* mutation, and overexpression of *AR* splice isoforms (Figure 3B). Another mechanism for increased AR signaling activity is the endogenous expression of androgen synthetic enzymes by tumor tissues, which leads to de novo androgen synthesis or conversion of weaker adrenal androgens into testosterone and DHT [124,211,212,213,214]. Up to 80% of CRPCs display a marked increase in AR mRNA and protein [215,216,217,218]. Studies by Kim et al. have shown that AR protein expression is increased in recurrent tumor samples compared to paired androgen-sensitive samples in tumor xenograft models [210]. Specifically, in CWR22 xenograft tumors, castration initially induced growth arrest in tumor cells. However, foci of Ki-67 immunopositive cells were detected by 120 days after castration [210]. In nearly one-third of patients progressing after castration or antiandrogen treatments, the mechanism for increased AR expression is through amplification of the *AR* gene at Xq11-12 [183,215,216,219,220,221]. Additionally, the most recent analysis of whole-exome sequencing of 150 metastatic CRPC (mCRPC) biopsies revealed 63% of *AR* gene amplification and mutation in comparison to that of 440 primary PCa tissues [222]. This amplification leads to an increase in *AR* gene expression and enhances AR activation by low levels of androgens. It remains unclear, however, whether amplification of the *AR* gene in hormone-refractory tumors results in an increase in AR protein levels. In fact, contradicting results have been obtained. Studies by Koivisto et al. have shown that hormone-refractory prostate tumors carrying an amplified *AR* express a higher level of *AR* mRNA compared to untreated primary tumors with a single copy of *AR* per cell [220]. In contrast, studies by Linja et al. have revealed that hormone-refractory tumors carrying *AR* amplification were not found to express a higher level of *AR* mRNA than those with a normal *AR* copy number [217]. Therefore, the significance of *AR* amplification in PCa remains unclear. In addition, alternative splicing of *AR* mRNA is another mechanism implicated in progression to CRPC. Multiple aberrantly spliced AR variants (ARV) that miss the C-terminal LBD were detected in CRPCs [222,223,224]. Importantly, all ARVs retain the amino-terminal transactivation and DNA-binding domains. AR-V7 (AR1/2/3/CE3 variant) is constitutively active and the most abundant variant detected to date in CRPC [225]. Interestingly, elevated AR-V7 induces expression of a unique set of target genes [225]. Furthermore, recent findings suggested that AR-V7 could have value as a predictive biomarker in CRPC. Antonarakis et al. showed that AR-V7 mRNA in circulating tumor cells (CTCs) might be enhanced by AR-directed therapies including abiraterone acetate and enzalutamide, and its expression was associated with poor prognosis [226]. Of note, the full-length AR and AR-Vs appear to almost always coexist in PCa cells; thus, it remains highly challenging to dissect their corresponding roles in driving AR signaling in translational studies of clinical specimens [183].

## 6. Interaction between AR and WNT Signaling in Prostate Cancer

The paradigm that PCa development and emergence of therapy resistance are a consequence of the restoration of embryonic developmental programs (e.g., WNT signaling) has shed a new light on understanding the molecular mechanisms underlying epithelial invasion in prostate development and development of CRPC. While the (aberrant) AR signaling pathway is considered as the most critical player in CRPCs, as intracellular signaling pathways are often interconnected, other pathways, in particular, the WNT pathway, can also play key roles. As noted in the previous section, considerable evidence indicates that the WNT pathway plays a central role in the development of prostate tissues, by providing developmental growth inductive signals during embryonic/neonatal organogenesis. In PCa, studies by Schaeffer et al. have reported that androgen exposure regulates genes previously implicated in prostate carcinogenesis; these genes included those related to developmental pathways, such as WNT signaling, along with cellular programs regulating such “hallmarks” of cancer as angiogenesis, apoptosis, migration and proliferation [227]. This observation was in line with the previously published data showing that aberrant activation of the WNT/β-catenin pathway contributes to progression of several other major human cancer types [27,30,35,56,90,100,228]. The prime example is colorectal cancer, in which approximately 85% of cases display *loss-of-function* mutations in the tumor suppressor *APC* gene [229,230,231,232]. APC protein recruits β-catenin to the degradation complex and its loss leads to upregulation of β-catenin signaling (Figure 1). In addition, mutations of serine/threonine residues within the N-terminal domain of β-catenin suppress β-catenin degradation, leading to constitutive activation of WNT signaling even in the absence of WNT ligands. In PCa, mutations in the *APC* or *CTNNB1* (β-catenin) genes, which lead to constitutive activation of WNT signaling, similar to those found in colon cancer, have also been identified [202,233,234,235,236].

**Figure 4:**
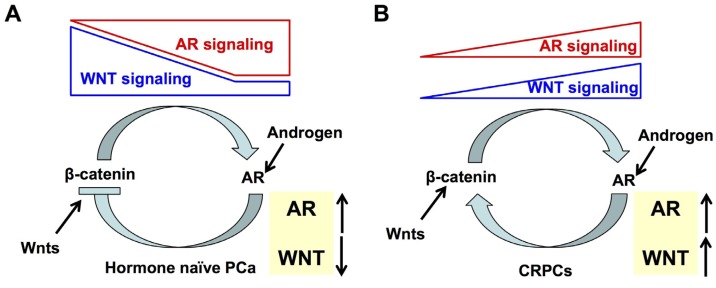
A simplified model of interaction between WNT and AR signaling during PCa development and progression. (**A**) In hormone naïve PCa cells, AR signaling inhibits the transcription of WNT/β-catenin target genes, while WNT/β-catenin signaling promotes transcription of AR target genes. Relative levels (i.e., anti-correlation but may reach to an equilibrium) of WNT (blue) and AR (red) signaling are indicated; (**B**) In CRPCs, AR and WNT/β-catenin signaling pathways stimulate each other to activate specific target genes for promoting androgen-independent growth and progression of PCa cells. Relative levels (i.e., positive correlation) of WNT (blue) and AR (red) signaling are indicated.

Accumulating evidence has supported that the WNT/β-catenin pathway plays an important role in CRPC, by interacting with AR signaling [234,237,238,239]. Several groups have focused on studying the role of β-catenin in CRPC compared to hormone-naïve PCa. Findings of a protein-protein interaction between AR and β-catenin have supported the biological significance of β-catenin in PCa cells. In 2000, Truica et al. showed that β-catenin could directly bind to AR to enhance its transcriptional activity stimulated by androgen, androstenedione, or estradiol, in LNCaP cells [240]. In 2002, Yang et al. demonstrated that β-catenin preferentially and directly bound to the LBD of AR in the presence of DHT over several other steroid hormone receptors [241]. Further studies revealed that β-catenin bound to the AF-2 region of the AR LBD, and modulated the transcriptional effects of the AR NTD as well as the p160 coactivator transcriptional intermediary factor 2 (TIF2); importantly, a single AR lysine residue (K720) has been shown to be necessary for the AR/β-catenin and TIF2/β-catenin interactions [242,243]. In β-catenin, early mapping experiments suggested that the NH2 terminus and the first six armadillo repeats of β-catenin were involved in its interaction with AR. In particular, deletion of repeat 6 fully abolished the physical interaction between AR and β-catenin, suggesting a key role of this repeat in the interaction [241]. Phenotypically, transient over-expression of β-catenin in AR^+^ PCa cell lines CWR22-Rv1 and LAPC-4 enhanced AR-mediated transcription of its target genes, in an androgen-dependent manner [244]. Hence, β-catenin (wild-type or mutated) is considered as a ligand-dependent co-activator of the AR-driven transcription (Figure 4). Binding of β-catenin to ligand-engaged AR also facilitates the movement of β-catenin into the nucleus [245]. Furthermore, it was shown that WNT/β-catenin signaling could increase *AR* gene expression via the TCF/LEF-1 binding sites in the *AR* promoter [246]. Thus, in hormone-naïve PCa, WNT/β-catenin signaling serves as a positive regulator of AR signaling in an androgen-dependent manner (Figure 4A).

In the other hand, the effect of AR signaling on WNT/β-catenin signaling is more complicated. Early studies in gonadotropin-releasing hormone neuronal cells showed that in the presence of DHT, liganded AR repressed β-catenin/TCF-responsive reporter gene activity [247]. In androgen-dependent LNCaP PCa cells, androgen treatment repressed target genes of WNT/β-catenin, whereas inhibition of AR activity enhanced WNT/β-catenin-responsive transcription; this data suggested that under the hormone-naïve condition, AR signaling could repress β-catenin/TCF-mediated transcription induced by androgen [96] (Figure 4A). Mechanistically, as β-catenin interacts with TCF4 to control transcription of WNT/β-catenin target genes, this could be due to preferential interaction of β-catenin with AR rather than TCF4 in hormone-naïve PCa cells. While WNT/β-catenin pathway is repressed by AR in the androgen-dependent LNCaP cell line, upon repression of AR activity or in the androgen-independent subline of LNCaP cells (LNCaP-abl), the WNT/β-catenin responsive transcription appeared to be largely activated, suggesting a likely role of WNT signaling in PCa progression to CRPC [96] (Figure 4B). This could be due to an increased interaction of β-catenin with TCF4 (rather than AR), which could promote WNT/β-catenin-target gene expression [96]. Therapeutically, pharmacological and genetic inhibition of the WNT/β-catenin pathway (using siRNA against β-catenin or a small molecule β-catenin inhibitor) in LNCaP-abl cells re-established their sensitivity to enzalutamide, a synthetic non-steroidal antiandrogen [96]. Thus, this study implies that inhibition of the WNT/β-catenin pathway may be translated into an effective therapeutic approach to treat enzalutamide-resistant CRPC.

To add another layer of the complexity of interaction between AR and WNT/β-catenin signaling, it was shown that when PCa cells had been adapted to the low androgen environment (e.g., upon ADT), β-catenin could act as a co-activator of AR as well to enhance AR transcriptional activity in the presence of androstenedione, a weaker adrenal androgen remaining present in CRPC patients [239,241,242,243]. This direct interaction between AR and β-catenin seemed to elicit a specific expression of a set of target genes in low androgen conditions in CRPC, which is consistent with the previous finding that target genes regulated by AR signaling are different in CRPC cells compared to those in hormone-naïve PCa cells [248]. Thus, it seems the effect of AR signaling on WNT/β-catenin signaling is PCa stage-dependent: it suppresses WNT/β-catenin signaling in hormone-naïve PCa, but in CRPC, both AR signaling and WNT/β-catenin signaling work together to positively support each other and to control a unique set of genes for sustaining CRPC cells (Figure 4). Lastly and most importantly, the significance of WNT/β-catenin and AR pathways in CRPCs was further demonstrated in studies by Robinson et al [222]. Their clinical sequencing analysis of PCa genomes has revealed that the majority of individuals with CRPCs harbor molecular alternations in the *AR* gene, as well as in genes encoding the main components of the WNT/β-catenin pathway, such as APC, β-catenin and R-spondins, leading to overactivation of WNT/β-catenin signaling [222].

As described in the previous section, WNT ligands are highly conserved secreted molecules that play critical but pleiotropic roles in cell-cell signaling during embryogenesis. Interestingly, expression levels of several WNT ligands were found to be up- or down-regulated in advanced PCa. For instance, Chen et al. demonstrated that high levels of WNT1 and β-catenin expression were associated with advanced, metastatic, hormone-refractory prostate carcinoma, in which they could serve as markers for disease progression [236]. In two independent studies, another WNT ligand, WNT3A, has been shown to modulate growth of PCa cells [20,249]. Importantly, the activity of AR signaling in the presence of low concentrations of androgens was increased by application of purified WNT3A, suggesting an important role of the canonical WNT3A signaling on the AR program [20]. As to the non-canonical WNT pathways, elevated levels of WNT5A have been found to increase free intracellular calcium and CaMKII in PCa cell lines, indicating that the WNT/Ca2+ pathway operates via CaMKII in PCa [250]. Yamamoto et al. showed that WNT5A overexpression enhanced invasion of the PC3 PCa cell line, and the invasion activity required the expression of WNT receptors FZD2 and ROR2 [251]. Interestingly, the very recent clinical studies by Miyamoto et al. have shown the importance of non-canonical WNT in the maintenance of metastatic CRPC [252]. In details, they used RNA-in-situ hybridization (RNA-ISH) to identify the source of WNT production in tumor specimens and CTCs. Metastatic tumor biopsies from patients with CRPC had readily detectable *WNT5A* and *WNT7B*. Similarly, *WNT5A* or *WNT7B* mRNA was detected by RNA-ISH in a subset of CTCs from patients with CRPC [252]. This demonstrates that a subset of PCa cells express non-canonical WNT ligands, which may provide survival signals in the context of AR inhibition. Furthermore, elevated expression of another WNT ligand, WNT11, has also been detected in PCa tissues versus normal samples [21]. Interestingly, WNT11 induced expression of neuroendocrine differentiation (NED) markers NSE and ASCL1, while silencing of WNT11 in androgen-depleted LNCaP and androgen-independent PC3 cells prevented NED and resulted in apoptosis [19].

Secreted WNT antagonists, including the sFRP family, DKK family, and Wnt inhibitory factor-1 (WIF1), are negative modulators of WNT signaling [239,253,254,255]. Thus, their expression is expected to be downregulated in advanced PCa. Indeed, a recent study reported downregulation of sFRP2 in PCa [256]. *WIF1* mRNA appears to be downregulated in a considerable percentage of PCa samples [257]. Interestingly, laboratories of Zi and Hoang have demonstrated that ectopic expression of sFRP3 (FRZB) or WIF1 in a CRPC cell line PC3 caused a reversal of epithelial-to-mesenchymal transition and inhibition of tumor growth by inhibition of the canonical WNT pathway [258,259]. The role of the DKK family of WNT antagonist (e.g., DKK1) in PCa is arguably even more complex than that of the sFRP family or WIF1. DKK1 inhibits WNT signaling by disrupting the binding of LRP6 to the WNT/FZD ligand-receptor complex [239,255]. Although DKK1 is upregulated in early PCa, it is downregulated during progression from primary tumor to metastasis; however, its expression can also inhibit WNT-induced osteoblastic activity and thus reduces bone metastases [260,261]. Altogether, these results suggest that WNT ligands and antagonists may play different roles during PCa progression in a context-dependent manner.

## 7. Therapeutic Applications for Targeting WNT/β-catenin-AR Interactions in CRPC

Cancer stem cells (CSCs) have been proposed to contribute to therapy resistance and cancer recurrence [262]. In addition to its higher activity in CRPC, the WNT/β-catenin signaling pathway has also been linked to prostate CSCs. For instance, Jiang et al. showed that activation of the WNT pathway via inhibition of GSK3β promoted LNCaP C4-2B and DU145 cell-derived xenograft tumor growth, as well as C4-2B cell-derived bone metastasis [263]. Interestingly, they reported an increase of the ALDH^+^/CD133^+^ CSC-like subpopulation in these PCa cell lines. Previous studies have shown that PCa cells with these markers exhibited tumor-initiating and metastasis-initiation cell properties, although it was not absolutely clear whether the ALDH^+^/CD133^+^ subpopulation represented CSCs definitively [263,264,265]. In a recent study [266], it was shown that knockdown of a prostate tumor suppressor, DAB2IP, transformed normal prostate epithelial cells into CSCs, which exhibited enriched CD44^+^/CD24^−^ populations. Interestingly, they reported that it was the WNT/β-catenin signaling pathway that mediated upregulation of CD44 by *DAB2IP* knockdown. In this setting, CD44 not only served as a marker for CSCs, but also played a key role in facilitating the onset of prostate CSCs and increasing their chemoresistance [266]. Importantly, combination therapy based on WNT inhibitors (e.g., LGK974) and conventional drugs (e.g., docetaxel) synergistically enhanced their efficacy and robustly inhibited growth of xenograft tumors [266]. In another study, Rajan et al. reported a gene expression profiling study of seven patients with advanced PCa, with paired samples before and after ADT [267]. By using RNA sequencing combined with bioinformatic approaches, the authors identified alterations in the WNT/β-catenin signaling pathway following ADT. Additionally, they showed that the tankyrase inhibitor XAV939 (which promotes β-catenin degradation) reduced growth of the androgen-independent LNCaP-abl cell line, compared with the androgen-responsive LNCaP cells [267]. Similarly, Lee et al. demonstrated that iCRT-3, a novel compound that disrupts both β-catenin/TCF and β-catenin/AR protein-protein interactions, inhibited PCa growth in vivo and blocked bicalutamide-resistant prostate sphere-forming cells [268]. Overall, it seems that targeting CSCs via inhibition of WNT signaling may have the potential to reduce the self-renewal and aggressive behavior of PCa [162].

As to the non-canonical WNT pathway, the most recent clinical studies by Miyamoto et al. have shown that activation of this pathway in CTCs from patients with metastatic CRPC correlates with reduced effectiveness of antiandrogen treatment [252]. In particular, significant enrichment of non-canonical WNT signaling was observed in CTCs from patients whose PCa progressed in the presence of enzalutamide, particularly among CTCs with reduced glucocorticoid receptor expression. To test whether activation of non-canonical WNT signaling modulates enzalutamide sensitivity, they ectopically expressed the ligands for non-canonical WNT signaling, including WNT4, WNT5A, WNT7B, or WNT11, in LNCaP PCa cells, which express these ligands at low endogenous levels. They found that ectopic expression of a range of these WNT proteins in androgen-sensitive LNCaP cells enhanced their survival in the presence of enzalutamide, with WNT5A to be particularly effective in this regard [252]. Conversely, its knockdown resulted in reduced cell proliferation. This data suggests that the non-canonical WNT signaling pathway may serve as a potential new therapeutic target in PCa that is resistant to antiandrogen therapy.

Taken together, WNT signaling interacts with AR signaling using distinct mechanisms at different stages of PCa progression. In hormone-naïve PCa cells, WNT/β-catenin signaling promotes transcription of AR target genes, whereas AR signaling inhibits the transcription of WNT/β-catenin target genes (Figure 4A). However, in CRPCs, the AR and WNT/β-catenin signaling pathways stimulate each other to activate a unique set of target genes for promoting androgen-independent growth and progression of PCa cells (Figure 4B). The interaction between AR and WNT signaling provides a growth advantage to PCa cells at the castration level of androgens. Inhibition of the WNT/β-catenin pathway would thus offer a novel therapeutic strategy to target CRPC cells and CSCs [239].

## 8. AR and WNT Signaling in Mammary Gland Development and Breast Cancer

### 8.1. AR and WNT Signaling in Mammary Gland Development

**Figure 5:**
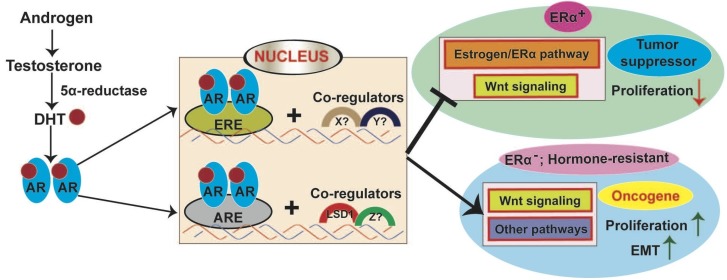
Proposed roles of AR and WNT signaling in mammary gland development and breast cancer. In breast cells, activated androgen/AR binds to ARE or ERE in its target genes. In ERα^+^ cells, it largely works as a tumor suppressor by inhibiting estrogen/ERα signaling and/or WNT signaling; in ERα^−^ cells or even in ERα^+^ cells that have become resistant to hormone therapy (targeting the estrogen/ER pathway), AR may function as an oncoprotein by activating WNT signaling and/or other oncogenic pathways. Under different cellular contexts, AR may utilize different co-regulators (e.g., LSD1, or other co-regulators remain to be defined (X?, Y?, or Z?)) to control distinct downstream programs.

WNT signaling plays key roles in both mammary gland development and breast cancer (BCa), largely through regulating mammary stem cell maintenance and basal mammary epithelial cell fate determination. An excellent review for this topic was published in this journal recently [41]. As to the AR signaling pathway, AR-mediated androgen actions play a direct or indirect role in mammary physiology (Figure 5). AR can interact with estrogen receptor alpha (ERα) and their interactions have inhibitory effects on their transactivational properties [269]. AR can also compete with ERα for binding to specific estrogen-responsive element (ERE) [270]. Thus, the effect of AR signaling in mammary gland development may be largely related to its effect on estrogen signaling. In fact, androgen treatment could inhibit estrogen-induced proliferation of mammary epithelial cells, particularly during puberty, leading to retarded mammary ductal extension and reduced expression of ERα [271,272,273]. Conversely, inactivation of AR resulted in accelerated mammary ductal growth and increased expression of ERα during puberty [273]. However, in addition to its inhibitory role on the ERα pathway, the role of AR signaling in mammary epithelial cells may be also mediated by inhibition of WNT/β-catenin signaling, a mechanism similar to that in hormone-naïve prostate cells (Figure 4). This is supported by the finding that loss of AR led to activation of the WNT/β-catenin pathway in the pubertal mammary gland [273]. In adult females, inhibition of AR signaling could also increase mammary ductal branching and mammary epithelial cell proliferation; however, this phenotype was not due to changes in serum estradiol levels or ERα expression, but was attributed to increased AR expression and consequently an increase in the ratio of AR to ERα (as ERα level remained constant) [271]. Relating to BCa, disruption of the inhibitory influence of androgen/AR signaling on mammary epithelial cells at either puberty or adult stage, as well as the crosstalk between AR signaling and estrogen or WNT signaling, are likely to have important implications for breast tumorigenesis [270,273].

### 8.2. AR Signaling in Breast Cancer

Unlike PCa, our understanding of AR signaling in BCa is still at its infancy. Some studies report that overexpression of AR is associated with better outcomes in BCa, while others illustrate a positive correlation of circulating androgens with high risk, recurrence and metastasis of BCa [274,275,276,277,278,279]. Historically, therapeutics targeting AR were considered beneficial for women diagnosed with advanced BCa [280]. In the “older generation” of androgen-related therapy for the treatment of BCa, including DHT, testosterone, and fluoxymesterone, certain clinical efficacies were observed [281,282,283]. However, androgen-related therapy gradually lost its attraction for the treatment of BCa, due to aromatization of androgens to estrogens, inconsistent clinical trials, undesirable virilizing side effects, and the broad utilization of estrogen-targeted therapy such as tamoxifen [284,285,286,287]. With improved preclinical interpretation of heterogeneity toward mammary epithelial cells and BCa subtypes, AR signaling-directed therapies, and resistance mechanisms of anti-estrogen therapies, there have been renewed enthusiasms in utilizing androgens and targeting AR for BCa [280,288].

In breast tissues, androgen can be converted to DHT, which subsequently activates AR. The liganded AR direct or indirectly (possibly together with distinct co-regulators under different ERα settings) interacts with either ARE or ERE in its target genes (Figure 5). In the presence of comparable levels of AR and ERα, AR competes with ERα, leading to inhibition of the estrogen/ER pathway [270,274]. In the absence of ERα (or under the conditional of resistance to hormone therapy), the ratio of AR to ERα increases and AR functions as an oncoprotein by recruiting different co-factors (e.g., lysine-specific demethylase 1 (LSD1)), leading to regulation of a different set of target genes, which may contribute to BCa cell proliferation and/or epithelial–mesenchymal transition (EMT) [270,289] (Figure 5).

BCa is often classified clinically into four subtypes based on expression of ER, progesterone receptor (PR), and human epidermal growth factor receptor 2 (HER2, also known as ERBB2): ER^+^/PR^+^/HER2^−^, ER^+^/PR^+^/HER2^+^, ER^−^/PR^−^/HER2^+^, and ER^−^/PR^−^/HER2^−^ (also known as triple negative breast cancer, TNBC). Relating to the ER status, AR likely plays distinct roles in BCa in a subtype-specific manner.

Positive expression of AR was clinically defined as immunohistochemical (IHC) nuclear staining ≥1% or ≥10% according to various studies [281,290,291,292]. AR is highly expressed in both primary (~80%) and metastatic (~60%) breast tumors [280]. AR expression varies in BCa across different subtypes; the prevalence of AR is approximately 70%–95%, 50%−81%, and 10%–53%, in ER^+^, ER^−^/HER2^+^, and TNBC subtypes, respectively [275,281,282,293,294,295,296,297,298].

Modulation of AR signaling, either inhibitory or stimulatory, exhibits somewhat contradictory observations in different subtypes of BCa, particularly when interacting with ER signaling [283,299]. When prescribed to non-selected BCa patients, testosterone contributed to a response rate of about 20%–25%; due to broad side effects, this strategy has quickly been replaced by multiple ER-directed therapies [300,301,302,303]. However, a retrospective study reported a promising tumor control rate of 58.5% (tumor regression and stableness, *n* = 53) with testosterone therapy in patients with metastatic ER^+^ BCa [304]. Androgen, together with tamoxifen, synergically increased response rates when treating advanced ER^+^ BCa, but this study is still at the beginning stage [280,305]. Recently developed AR antagonists have demonstrated more potent and better clinical efficacies than those of the early-generations, which have generally been disappointing for combating BCa [280,288,306,307]. Here we will highlight the key AR-based therapeutics for treatment of BCa, in a subtype-specific manner.

#### 8.2.1. AR in ER^+^ Breast Cancer

AR is highly expressed in ER^+^ BCa with a frequency of ~70%–95% [281,295,296,298,308]. In this BCa subtype, ER signaling functions as a dominant oncogenic driver; thus, clarifying its functional relationship with AR signaling would be beneficial for exploring the role of anti-estrogen therapies [309]. AR and ER can interact (and interfere) with each other functionally by sharing (and competing for) similar cofactors and nuclear binding sites [274,310]. AR expression may have contradicting functional consequences in ER^+^ BCa in a treatment-dependent manner: some studies indicated that higher AR expression is associated with better therapy outcomes, whereas others have reported that AR plays an oncogenic role in tamoxifen-resistant subjects [294,311,312,313,314]. Nevertheless, AR signaling may mainly play an anti-proliferative effect in ER^+^ BCa initially, due to its ability to antagonize the growth-promoting role of ER signaling [302]. Accordingly, androgens and androgen agonists have been evaluated for the efficacies of treating ER^+^/AR^+^ BCas [302]. But combination therapy based on enzalutamide (antiandrogen) and agents that target ER signaling (e.g., exemestane, anastrozole, or fulvestrant) has also been tested in clinical trials for potentially overcoming resistance to hormone therapy [294].

#### 8.2.2. AR Signaling in ER^−^/HER2^+^ Breast Cancer

AR is highly expressed in ER^−^ BCa and the functional crosstalk between AR and HER2 is critical for the tumor cell survival and expansion [282,297,315]. In this subtype of BCa, the proliferative role of AR signaling has been well investigated [275,280]. Mechanisms underlying this functional interplay include direct transcriptional upregulation of HER2 signaling by AR via its heterodimer HER3, which in turn activates *AR* transcription in a positive feedback loop [297,316,317]. AR signaling also induces ligand-dependent stimulation of WNT signaling, via direct transcriptional upregulation of *WNT7B,* which activates β-catenin, resulting in *HER3* transcriptional activation [297]. HER2 signaling is the key oncogenic driver in this subtype of BCa and effective HER2-targeted therapies are crucial for treating patients with this BCa subtype. As AR antagonists can efficiently reduce cell proliferation [297,318], clinical trials are ongoing to explore whether combination of AR and HER2-directed therapies could result in any synergic outcomes [318].

#### 8.2.3. AR Signaling in TNBC

The frequency of AR expression in TNBC is around 10% to 53% [281,296,298]. A molecular subtype of BCa referred to as the molecular apocrine subtype, which included those non-basal-like ER^−^ breast tumors that were also AR^+^, was defined based on microarray expression profiling [319]. Later on, also based on gene expression profiling data, TNBCs were classified as six subtypes and those with AR expression were defined as the luminal androgen receptor (LAR) subtype [298]. Differentially expressed genes that characterize this subtype are heavily enriched in hormonally regulated pathways, including steroid synthesis, porphyrin metabolism, and androgen/estrogen metabolism [298,320]. AR signaling in TNBC was reported to maintain cell proliferation and AR also acted as a biomarker for sensitivity to both PI3K and ERK inhibition [318,321]. The functional role of AR in TNBC was further established based on the finding that LAR BCa cells were sensitive to AR antagonists and Hsp90 inhibitors [322]. An encouraging case for using AR-targeted therapy for treatment of AR^+^ TNBC was reported recently, in which a patient with this BCa subtype had progressive disease following six cycles of cytotoxic chemotherapy, but attained a 100% response to bicalutamide (an antiandrogen) [323]. With the development of potent AR-directed therapies and promising combined therapeutic approaches, more clinical trials targeting AR^+^ TNBC are being developed [318,321].

### 8.3. Interaction between AR and WNT Signaling in Breast Cancer

Overexpression of WNT induces aberrant activities of the WNT signaling pathway, which is a main driving force in BCa progression [297,324]. WNT ligands are associated with normal mammary gland development and overexpression of *WNT1* is oncogenic for BCa [325]. The interplay of AR and WNT signaling has been mainly studied in the ER^−^/HER2^+^ BCa subtype. Using gene set enrichment analysis (GSEA), Ni et al. observed that androgen (DHT)-stimulated genes in ER^−^/HER2^+^ BCa cells were mainly those involved in WNT signaling [297]. Furthermore, they found that AR upregulated *WNT7B* transcription in a ligand-dependent manner. WNT7B is a canonical WNT ligand and may play roles in the normal mammary gland development during the stages of ductal formation and involution [326,327]. Elevated expression of *WNT7B* has been found in ~10% of BCa cases [328]. In addition to activation of WNT signaling via the androgen/AR-WNT7B pathway, Ni et al. showed that similar to PCa, AR and WNT/β-catenin signaling also cooperated functionally; in this case, β-catenin cooperated with AR to promote the progression and maintenance of ER^−^/HER2^+^ BCa cells by upregulating *HER3*, which encodes a key co-receptor of HER2 in HER2^+^ BCa [297]. Importantly, by targeting the AR pathway using bicalutamide, the growth of DHT-stimulated ER^−^/HER2^+^ breast tumor cells in vivo was inhibited [297].

Thus, in both PCa and BCa, AR signaling appears to regulate distinct sets of target genes in hormone-dependent cancers (i.e., hormone-naïve PCa, ER^+^ BCa) and hormone-refractory cancers (i.e., CRPC, ER^−^ BCa, hormone therapy-resistant ER^+^ BCa). Accordingly, both AR agonists and AR (and/or WNT) antagonists may be beneficial for BCa therapy, but in a BCa subtype and therapy stage-dependent manner. In particular, as both the AR and WNT signaling pathways drive progression and maintenance of AR^+^ TNBCs, inhibitors for these two pathways may prove to be useful for targeting this TNBC subtype. In addition, AR antagonists and anti-HER2 agents may also be used in combination to treat ER^−^/HER2^+^ BCa with AR expression, and inhibitors for WNT signaling may offer another therapeutic opportunity, particularly when ER^−^/HER2^+^ BCa cells develop resistance to the anti-HER2/AR agents.

## 9. Concluding Remarks

As two key pathways regulating both normal development and tumorigenesis in hormone-responsive prostate and mammary glands, the context-dependent interplay of AR and WNT signaling pathways provides a unique opportunity to explore therapeutic options for treating prostate and breast cancers, particularly when under the setting of therapeutic resistance. As both CRPCs and ER^−^ BCas (i.e., TNBC and ER^−^/HER2^+^ BCa, or even ER^+^ BCas that become resistant to hormone therapy) are refractory or unresponsive to hormone therapy, a better understanding of roles of AR and WNT pathways and their interactions in these hormone-refractory diseases should open a new avenue for improving their treatment and for combating the inevitable challenge of therapy resistance.
